# Nitric oxide is not responsible for initial sensory-induced neurovascular coupling response in the barrel cortex of lightly anesthetized mice

**DOI:** 10.1117/1.NPh.12.S2.S22802

**Published:** 2025-06-20

**Authors:** Llywelyn Lee, Luke W. Boorman, Emily Glendenning, Changlin Shen, Jason Berwick, Clare Howarth

**Affiliations:** a University of Sheffield, School of Psychology, Sheffield, United Kingdom; b University of Sheffield, Neuroscience Institute, Sheffield, United Kingdom; c University of Sheffield, Healthy Lifespan Institute (HELSI), Sheffield, United Kingdom

**Keywords:** nitric oxide, neuronal nitric oxide synthase interneuron, neurovascular coupling, vasomotion, inhibitory interneuron, optogenetics

## Abstract

**Significance:**

Neurovascular coupling matches changes in neural activity to localized changes in cerebral blood flow. Although much is known about the role of excitatory neurons in neurovascular coupling, that of inhibitory interneurons is unresolved. Although neuronal nitric oxide synthase (nNOS)-expressing interneurons are capable of eliciting vasodilation, the role of nitric oxide in neurovascular coupling is debated.

**Aim:**

We investigated the role of nitric oxide in hemodynamic responses evoked by nNOS-expressing interneurons and whisker stimulation in mouse sensory cortex.

**Approach:**

In lightly anesthetized mice expressing channelrhodopsin-2 in nNOS-interneurons, 2D optical imaging spectroscopy was applied to measure stimulation-evoked cortical hemodynamic responses. To investigate the underlying vasodilatory pathways involved, the effects of pharmacological inhibitors of NOS and 20-HETE were assessed.

**Results:**

Hemodynamic responses evoked by nNOS-expressing interneurons were altered in the presence of the NOS inhibitor LNAME, revealing an initial 20-HETE-dependent vasoconstriction. By contrast, the initial sensory-evoked hemodynamic response was largely unchanged.

**Conclusions:**

Our results challenge the involvement of nNOS-expressing interneurons and nitric oxide in the initiation of functional hyperemia, suggesting that nitric oxide may be involved in the recovery, rather than initiation, of sensory-induced hemodynamic responses.

## 1 Introduction

The ability to regulate cerebral blood flow (CBF) in a localized and dynamic manner in response to neuronal activity is essential for the maintenance of healthy brain function. Although the regulation of CBF by glutamatergic neurons has been well investigated,^1^ regulation by GABAergic inhibitory interneurons (INs) has received less attention. Using optogenetic approaches to specifically activate distinct neural populations, recent research has revealed not only that cortical INs are capable of regulating CBF^2^^–^^4^ but also that subpopulations of cortical INs, which release different vasoactive molecules,^5^ can drive distinct hemodynamic responses.^6^^–^^9^ The vasodilatory pathways involved in specific IN-evoked hemodynamic responses remain understudied. Understanding how, and when, INs regulate CBF would inform our interpretation of functional imaging signals such as blood oxygen level-dependent functional magnetic resonance imaging (BOLD fMRI), in which blood-based signals act as a proxy for neural activity.^10^ Furthermore, understanding IN regulation of CBF has relevance for conditions in which altered CBF and IN dysfunction have previously been reported, such as Alzheimer’s disease,^11^^–^^13^ epilepsy,^14^^,^^15^ and ageing.^16^^,^^17^

Having previously demonstrated that neuronal nitric oxide synthase-expressing interneurons (nNOS INs) are able to evoke robust changes in cerebral blood volume and oxygenation,^7^ in this study, we aimed to investigate the vasodilatory pathways underlying nNOS IN-evoked hemodynamic changes. A prime candidate was nitric oxide (NO), which is produced by three isoforms of the enzyme nitric oxide synthase (NOS): neuronal NOS (nNOS), expressed in neurons; endothelial NOS (eNOS), expressed in vascular endothelial cells; and inducible NOS (iNOS), expressed during inflammatory responses.^18^ NO release by nNOS INs (which target blood vessels^19^^,^^20^) may also underpin CBF responses evoked by VGAT-expressing INs.^4^ Furthermore, NO is involved in maintaining basal vascular tone^21^ and has been suggested as a key mediator in sensory-evoked neurovascular coupling (as reviewed in Ref. 22). NO can evoke vasodilation via several vasoactive pathways. In addition to relaxing smooth muscle cells via a cGMP-associated pathway,^23^ NO can also inhibit the production of 20-hydroxyeicosatetraenoic acid (20-HETE^24^), a vasoconstrictor,^25^^–^^27^ thus enhancing vasodilation.^28^^–^^30^ However, the role of NO in neurovascular coupling remains unresolved, with genetic or pharmacological inhibition of NOS reported to decrease,^29^^,^^31^^–^^34^ increase,^31^^,^^35^ and have no effect on^4^^,^^29^^,^^31^^,^^36^^–^^39^ hemodynamic responses to sensory stimulation.

Therefore, this study investigated the involvement of NO in nNOS IN-evoked and sensory stimulation-evoked hemodynamic changes. To this end, in whisker barrel cortex of lightly anesthetized mice, we recorded hemodynamic responses to short duration (2 s) optogenetic activation of nNOS INs and whisker stimulation, in the presence and absence of the pharmacological non-selective NOS inhibitor N-nitro-l-arginine-methyl-ester (LNAME), which suppresses NO production. We report that although NO is involved in the initial hemodynamic response to nNOS IN activation, counteracting the constrictive effects of 20-HETE release, NO is not involved in the initiation of sensory-evoked hemodynamic responses but does alter return to baseline dynamics. The observed effect on post-stimulus recovery may be due to an interaction with LNAME-evoked vascular oscillations.

## 2 Materials and Methods

### 2.1 Animals

All procedures involving animals were performed in accordance with the UK Government, Animals (Scientific Procedures) Act 1986, were approved by the University of Sheffield ethical review and licensing committee, and were reported in line with the ARRIVE guidelines.^40^ Mice had ad libitum access to food and water and were housed on a 12 h dark/light cycle. A total of 23 mice were used (14 males and 9 females), aged 4 to 9 months old (6.58±1.36 months) and weighing 20 to 36 g (29.08±5.73  g) on date of surgery. Mice were nNOS-CreERT × ChR2-EYFP (nNOS-ChR2), obtained by crossing heterozygous nNOS-CreERT mice (Stock 014541, Jackson Laboratory, Bar Harbor, Maine, United States^41^) with homozygous ChR2(H134R)-EYFP mice (Stock 024109, Jackson Laboratory^42^), as used previously.^7^ Mice positive for the nNOS-CreERT insertion (confirmed by genotyping of ear clips from pups) were used in these experiments. ChR2 expression was induced by intraperitoneal (i.p.) injection of tamoxifen (Sigma-Aldrich, St. Louis, Missouri, United States) at 100  mg/kg, administered three times with a day between each injection. Treatment with tamoxifen was carried out when mice were aged between 1 and 5 months and took place a minimum of 2 weeks prior to surgery to allow for gene expression to occur. Randomization sequences were not used to assign animals to different pharmacological agents.

### 2.2 Surgical Preparation of Chronic Cranial Window

At least 2 weeks prior to experimental sessions, surgery to prepare a thinned cranial window over the right somatosensory cortex was performed as previously described by Sharp et al.^43^ In brief, i.p. injection of fentanyl-fluanisone (Hypnorm, Vetapharma Ltd., Leeds, UK), midazolam (Hypnovel, Roche Ltd., Basel, Switzerland), and sterile water (in a ratio of 1:1:2 by volume; 7  mL/kg) were used to induce anesthesia, and anesthesia was maintained using isoflurane (0.5% to 0.8%) in 100% oxygen at a flow rate of 1  L/min. Surgical anesthetic plane was monitored by regularly checking toe-pinch reflex response. To avoid optogenetic activation of ChR2, surgeries were performed in a dark room using a surgical light with a band pass filter (577±5  nm). Mice were placed in a stereotaxic frame (Kopf Instruments, Tujunga, California, United States) with a homoeothermic blanket (Harvard Apparatus, Holliston, Massachusetts, United States) maintaining rectal temperature at 37°C. Using a dental drill, an area of bone (∼3  mm×3  mm) overlying the right whisker barrel cortex was thinned to translucency, and a thin layer of clear cyanoacrylate was applied to smooth and reinforce the area. Dental cement (Super bond C&B, Sun Medical, Shiga, Japan) was used around the window to secure a stainless steel head-plate to the skull. Following surgery, mice were singly housed and were monitored using the mouse grimace scale^44^ and weighed weekly. Any animals losing over 20% body weight post-operatively were culled; for this study, no mice met these criteria.

### 2.3 2D Optical Imaging Spectroscopy

Anesthesia was induced via i.p. injection of fentanyl-fluanisone (Hypnorm, VetaPharma Ltd), midazolam (Hypnovel, Roche Ltd), and sterile water (in a ratio of 1:1:2 by volume; 7  mL/kg), and anesthesia was maintained using isofluorane (0.25% to 0.7%) with 100% oxygen at a flow rate of 0.8  L/min. Mice were head fixed within a stereotaxic frame, using the head-plate that was attached during surgery. A homeothermic blanket maintained rectal temperature of the mouse at 37°C. 2D Optical Imaging Spectroscopy (2D-OIS) allows changes in cortical hemodynamics (oxygenated hemoglobin: Hbo, deoxygenated hemoglobin: Hbr, and total hemoglobin: Hbt) to be measured. 2D-OIS was performed using our previously published methodology.^7^ In brief, using a Lambda DG-4 high-speed filter changer (Sutter Instrument Company, Novato, California, United States), the cortex under the thinned window was illuminated with four wavelengths of light (587±9  nm, 595±5  nm, 560±15  nm, 575±5  nm). The remitted light was collected with a Dalso 1M60 CCD camera with a frame rate of 32 Hz. This was synchronized to the wavelength switching, resulting in an effective frame rate of 8 Hz. To avoid collecting light from the 470 nm photostimulation LED, the camera was fitted with a 490 nm high pass filter. Spectral analysis was carried out pixel-by-pixel on the remitted light, based on the path length scaling algorithm (PLSA) as described previously,^45^^,^^46^ which allowed the generation of estimates for the changes from baseline of Hbo, Hbr, and Hbt. The analysis assumed a baseline tissue concentration of hemoglobin of 100  μM and oxygen saturation of 70%. This spectral analysis resulted in the production of 2D spatial images representing micromolar changes in the concentration of Hbo, Hbr, and Hbt, over the time course of an experiment.

### 2.4 Electrophysiology

In some experiments neural activity was recorded concurrently with hemodynamic activity, to effectively assess both these aspects of neurovascular coupling. A cranial burr-hole was made in the right whisker barrel cortex (identified by response to whisker stimulation in a previous 2D-OIS experiment) to allow insertion of a 16 channel microelectrode (100  μm spacing, 1.5 to 2.7 Ω impedance, site area $177\;\mu m^{2}$; Neuronexus Technologies, Ann Arbor, Michigan, United States) to a depth of ∼1600  μm. To record neural activity, the electrode was connected to a preamplifier and data acquisition device (Medusa BioAmp/RZ5, TDT), and data were collected at 24 kHz. During post-hoc analysis, data were downsampled to 6 kHz, and a 500 Hz high pass filter was applied. A “spike” was detected when data exceeded a threshold of 1.5 times the standard deviation above the mean. The number of spikes occurring in 100 ms bins were counted and reported as MUA. Data from the 12 electrode channels corresponding to cortical depth were used, with data from channels 3 to 6 being used for time series analysis. To produce fractional change in MUA, responses were normalized to a 2 s baseline. Trials were averaged to produce a mean response for each stimulation paradigm for each animal. For statistical analysis, the peak MUA and mean MUA during the stimulation period were calculated for each animal. Group means were produced by averaging across animals. One mouse was excluded from statistical analysis due to the presence of excessive noise.

### 2.5 Stimulations

#### 2.5.1 Photostimulation

A fiber-coupled 470 nm LED light source (ThorLabs, Newton, New Jersey, United States) connected to a fiber optic cable (core diameter 200  μm, Thorlabs) delivered blue light for photostimulation of ChR2. 2s light delivery consisted of 10 ms pulses at 99 Hz (1 V, 0.45 mW). We have previously confirmed that these photostimulation parameters do not elicit hemodynamic responses in non-ChR2-expressing mice.^7^

#### 2.5.2 Whisker stimulation

A plastic T-bar attached to a stepper motor moved whiskers of the left whisker pad ∼1  cm in the rostro-caudal direction.^43^ Whiskers were stimulated for 2 s, at 5 Hz.

#### 2.5.3 Simultaneous presentation

Simultaneous presentation of photostimulation and whisker stimulation was applied to assess whether the evoked hemodynamic responses summed in a linear manner.

To mitigate effects of time since anesthesia or pharmacological treatment, optogenetic, whisker, and simultaneous stimulations were interleaved within each experiment. To improve the signal to noise ratio, each experiment contained 20 to 30 repeats of each stimulation type, with an inter-stimulus interval (ISI) of 25s.

### 2.6 Pharmacology

To reduce NO production, mice were treated with the nonselective NOS inhibitor N(G)-Nitro-l-arginine methyl ester (LNAME, Sigma). LNAME (10  mg/mL made up with sterile saline) was administered via i.p. bolus injection (75  mg/kg; which has been shown to reduce NOS activity within the cortex by 93% within 1 h of injection^47^). 20-HETE production was inhibited by treatment with a selective inhibitor of CYP4A and 4F, N-(4-butyl-2-methylphenyl)-N′-hydroxy-methanimidamide (HET0016).^48^ HET0016 (Santa Cruz Biotechnology, Dallas, Texas, United States) was administered i.v. (tail vein, 10  mg/kg; a 5  mg/mL solution was made up with 10% lecithin saline^49^^,^^50^).

Three cohorts of mice were used in these studies: 12 mice received LNAME alone, 5 mice received HET0016 alone, and 6 mice received LNAME and HET0016 together.

### 2.7 Procedure

Following anaesthetic induction, an electrode was inserted into the right whisker barrel cortex (if needed). 2D-OIS measurements of hemodynamic changes evoked by stimulation (photostimulation and/or whisker stimulation) were monitored continuously. Injection of pharmacological agent(s) occurred ∼1  h after 2D-OIS experiments started which, in the case of electrode insertion, allowed sufficient time for hemodynamics to recover from the resulting cortical spreading depolarization (CSD).^51^ Timings of agent injection were kept consistent, regardless of whether an electrode was inserted. For this study, data from specific timepoints pre- and post-treatment were analyzed. Pre-treatment responses to stimulation were taken from the experiment immediately prior to the injection of pharmacological agent(s). Responses to stimulation post-treatment were taken from the experiment occurring at the following time after injection of pharmacological agent(s): LNAME: 70 to 135 min (mean=108±7  min) after injection which, in agreement with previous studies,^47^^,^^52^ was sufficient time for LNAME to have maximal effect (Fig. 2); HET0016: 70 to 145 min (mean=125±12  min); LNAME and HET0016: 70 to 135 min (121±9  min) after injection; No inhibitor (timing control experiments): 80 to 120 min (100±6  min) after the “injection” timepoint.

### 2.8 Data Analysis

**Fig. 1:**
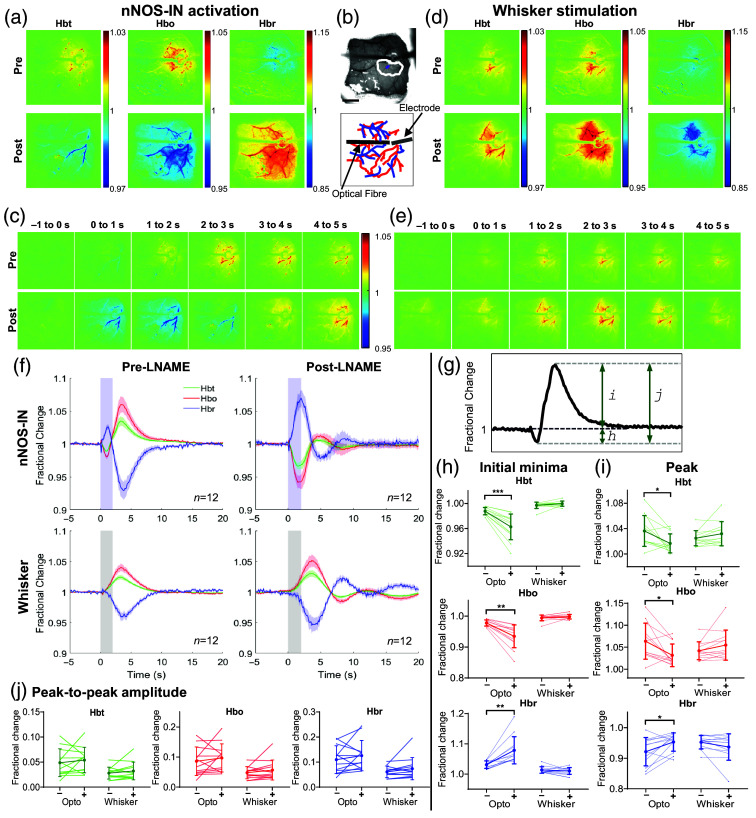
NOS-dependence of hemodynamic responses evoked by 2s nNOS-IN activation or whisker stimulation. Data from representative mouse showing hemodynamic response to 2s optogenetic stimulation of nNOS-INs (a)–(c) or 2 s whisker stimulation (d)–(e). (a), (d) Trial-averaged stimulation-evoked changes in Hbt, Hbo, and Hbr, compared with the baseline, pre-LNAME (upper) and post-LNAME (lower) injection. Color bars represent fractional change. (b): Thinned cranial window (upper) with cortical surface vasculature visible (imaged at 575 nm illumination), optic fiber and electrode can be seen. White ROI indicates whisker barrel cortex and purple ROI indicates arteriole region, from which timeseries data are extracted. Scalebar represents 1 mm. Diagram (lower) indicating surface arteries (red) and veins (blue) visible through thinned skull window. (c), (e) Evolution of trial-averaged changes in Hbt. Stimulation of nNOS-INs (c) or whiskers (e) occurs at 0 to 2s. Color bar represents fractional change. Time points relative to stimulation onset (0 s) are indicated above the images. (f) Group data (n=12 mice). Mean fractional change in Hbt, Hbo, and Hbr in arteriolar ROI before (left) and after (right) LNAME. Blue shading indicates photostimulation period (upper), grey shading indicates whisker stimulation period (lower). Data: mean ± SEM (g) Hbt time series from example mouse illustrating three metrics used for analysis: initial minima (h), peak (i), peak-to-peak amplitude (change between peak and initial minima), (j). (h)–(j) Fractional change in Hbt, Hbo, and Hbr in response to optogenetic and whisker stimulation with (+) and without (−) LNAME. Darker lines represent group mean ± *SD*, lighter lines indicate trial-averaged mean for individual animals. n=12 mice. *p<0.025, **p<0.005, ***p<0.0005. (h) Initial fractional change (“initial minima”). (i) Peak fractional change. (j) Maximum stimulation-evoked net change (minima to maxima).

All experiments and analysis were performed unblinded. Data analysis was performed using MATLAB (MathWorks, Natick, Massachusetts, United States). Using the spatial map of Hbt changes evoked by 2 s whisker stimulation, generated by 2D-OIS, a region of interest (ROI) was automatically selected.^7^ In brief, each pixel was averaged across time during the response period, to generate a mean pixel value. Any pixel with a value greater than 1.5× standard deviation was included in the ROI. The resulting ROI [white ROI, Fig. 1(b)] represents the area with the greatest hemodynamic response to the whisker stimulation and therefore represents the whisker barrel cortex. Within this ROI, the arterial region most responsive to the pharmacological intervention was manually selected [purple ROI, Fig 1(b)]. For each stimulation paradigm, the response across all pixels within the arterial ROI was averaged, producing the three hemodynamic time series (i.e., Hbo, Hbr, and Hbt).

#### 2.8.1 Baseline assumptions

For spectral analysis within the arterial ROI, before pharmacological intervention, a baseline oxygen saturation of 80% (in line with previously published values for anesthetized mice with supplemental oxygen^53^^,^^54^) and tissue concentration of hemoglobin of 100  μM were assumed.^55^^,^^56^ Pharmacological agents, including LNAME,^57^^,^^58^ have previously been shown to alter myogenic tone. As data were continuously collected during our experiments, it was possible, for each mouse, to measure the change in Hbt and Hbo evoked by treatment with pharmacological agents. Although these changes were minor, baseline assumptions for spectral analysis at post-pharmacological treatment periods were individually amended accordingly (see Table S1 in the Supplementary Material for group mean values).

#### 2.8.2 Comparison of stimulation-evoked responses

To compare stimulation-evoked responses in trials involving photostimulation, residual artefact from the 470 nm LED was removed using a modified boxcar function. For all stimulations, micromolar changes in hemodynamics were converted to fractional change (compared with a 5 s period before stimulation), ensuring that the results were less sensitive to our baseline assumptions.^59^ For each stimulation paradigm, mean time series were produced for each animal by averaging across trials. To produce the group mean time series, these responses were then averaged across animals within each group.

#### 2.8.3 Low-frequency vascular oscillations

The last 1000 s of the Hbt timeseries of “pre-” and “post-” experiments were used to assess low-frequency vascular oscillations. A cubic polynomial trend was removed from the data, and the amplitude of the signal was normalized between 0 and 1, before computing the Fourier transform (FFT). The area under the curve (AUC) between 0.09 and 0.11 Hz was calculated to compare oscillations centered around 0.1 Hz (vasomotion). To produce the group mean, FFTs (and associated AUC) were averaged across animals within each group.

For visualization purposes [Figs. 7(a) and 7(c)], following the removal of the cubic polynomial trend, a first-order Savitzky–Golay filter was applied to the individual Hbt time series to smooth high-frequency components (e.g., heart rate).

### 2.9 Statistics and Reproducibility

Three metrics [Fig. 1(g)] were extracted from the stimulation-evoked hemodynamic time series: initial minima: the minimum value of Hbt and Hbo and maximum value of Hbr during the initial response period (defined as 0.25 to 5 s after stimulation onset); peak: the maximum value of Hbt and Hbo and minimum value of Hbr during the response period (defined as 0.25 to 10s after stimulation onset); and peak-to-peak amplitude: the change between peak and trough (minima) in Hbt, Hbo or Hbr ([peak] – [minima]). Statistical analysis was carried out using SPSS (versions 26 and 28). Paired dot plots and violin plots were prepared using GraphPad Prism (version 9.3.1 and 10.4.2, respectively). Shapiro–Wilk test was used to assess normality of data (ANOVA was considered robust if data were approximately normally distributed), and Levene’s test was used to test for equality of variances. Outliers were identified as extreme if they had a studentized residual >3. For experiments in which LNAME was applied alone, to determine statistical significance, a three-way mixed ANOVA (within-group factors: drug [pre/post], stimulation type [photostimulation/whisker]; between group factor: electrode [absent/present]) was used. For other pharmacological agents and MUA, a repeated measure two-way ANOVA was used (factors: drug [pre/post], stimulation type [photostimulation/whisker]). To assess differences in arterial oscillations, spectral power (AUC) was compared using a mixed two-way ANOVA (between-group factor: drug [LNAME/no drug]; within-group factor: time [pre/post agent application]). In the spectral power comparison, one data point was identified as an extreme outlier; however, its inclusion did not alter the outcome of the statistical analysis, and so, all data were included. For all multiway ANOVAs, simple effects tests were carried out to further interrogate any significant interaction effects. To assess the evolution of the effect of LNAME, three timepoints (pre-LNAME injection, 0 min after LNAME injection, and 60 to 70 min after LNAME injection) were compared using a one-way ANOVA with Greenhouse–Geisser correction applied. A paired two-tailed t-test was used to compare temporal characteristics (rise time [time from 10 to 85% of peak response], time to peak, and onset time) of the whisker stimulation-evoked hemodynamic response before and after LNAME and to assess whether there were sex-dependent differences. For cases in which Shapiro–Wilks test suggested that data were not normally distributed, a Mann–Whitney U test was used to compare groups. The results were considered statistically significant if p<0.05, unless otherwise stated. All data are reported as mean ± standard error of the mean (SEM), n = number of mice, unless otherwise stated. Sample sizes were based on those in previously published studies using similar pharmacological approaches.^47^

## 3 Results

### 3.1 NO is Involved in the Initial nNOS IN-Evoked Hemodynamic Response But Not in the Initiation of a Sensory-Evoked Hemodynamic Response

Genetically modified mice expressing channelrhodopsin-2 (ChR2) in nNOS INs were used to investigate the involvement of NO in nNOS IN- and sensory-evoked cortical hemodynamic responses. In this mouse line, we previously observed that ChR2 expression occurs in ∼90% of nNOS INs^7^ with a selectivity of 94.7% (248/262 cells, 3 mice, 3 sections from each mouse^7^). Thus, blue light can be used to selectively activate nNOS INs. Here, in lightly anaesthetized mice, we combined 2D optical imaging spectroscopy (2D-OIS), optogenetics, and pharmacological blockade to assess whether the localized hemodynamic response evoked by short duration (2s) nNOS IN activation or sensory stimulation was dependent on NO produced by NOS. High-resolution 2D maps of stimulation-evoked changes in blood volume (Hbt), oxygenated hemoglobin (Hbo) and deoxygenated hemoglobin (Hbr) were recorded before and after treatment with LNAME (75  mg/kg, i.p.^47^). Mice first received a short duration (2 s) mechanical stimulation of the whiskers to define the whisker barrel cortex and guide placement of the optrode and, where appropriate, multichannel electrode. The optrode, consisting of a fiber optic-coupled blue LED (470 nm) placed directly above the center of the whisker barrel cortex, delivered the photostimulation necessary to activate ChR2. Each animal received interleaved whisker stimulations (2 s, 5 Hz), photostimulations (2 s, 99 Hz) and, in a subset of animals, simultaneous photostimulation and whisker stimulation. The resulting hemodynamic changes were centered around the optrode [photostimulation: Fig. 1(a)] and localized to the whisker barrel cortex [whisker stimulation: Fig. 1(d)], as we have previously reported.^7^ Prior to treatment with LNAME, both 2 s photostimulation of cortical nNOS INs [Figs. 1(a) and 1(c), top rows] and sensory stimulation [Figs. 1(d) and 1(e), top rows] elicited localized increases in concentration of Hbt and Hbo and decreases in Hbr concentration. As previously described,^7^ the largest increases in Hbt and Hbo were observed in branches of the middle cerebral artery (MCA) overlying the whisker barrel cortex, and a decrease in Hbr was observed in the draining veins [Figs. 1(a) and 1(d), top row]. In response to photostimulation of nNOS INs, the time series of the hemodynamic response taken from an arterial region of interest [ROI, purple ROI Fig. 1(b)] revealed a bidirectional response comprising of an initial decrease in Hbt and Hbo accompanied by a concomitant increase in Hbr (“initial minima”), followed by an increase in Hbt and Hbo and decrease in Hbr (“peak”), which peaked following the cessation of stimulation [Figs. 1(f), top left]. By contrast, the time series of the hemodynamic response to whisker stimulation consisted solely of an increase in Hbt and Hbo and corresponding washout of Hbr, which peaked following the cessation of stimulation [“peak,” Figs 1(f), bottom left], with no measurable “initial minima.”

**Fig. 2:**
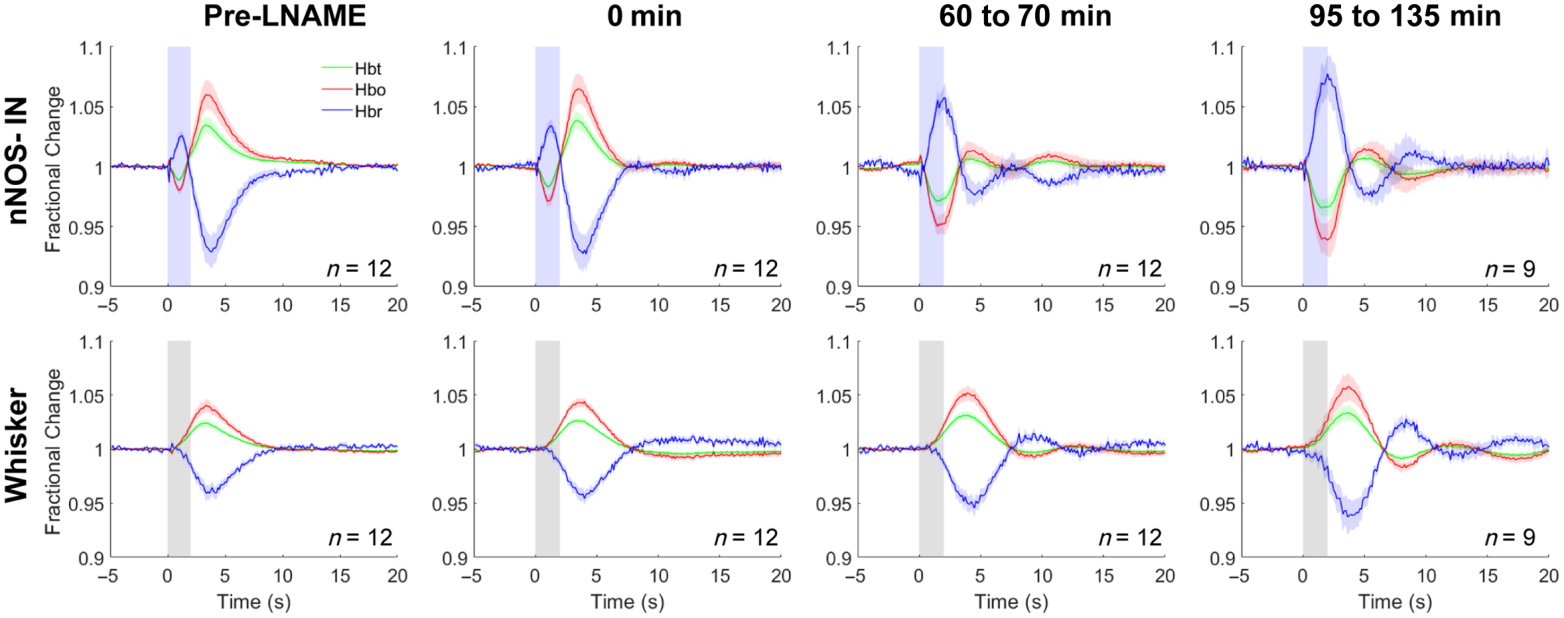
Development of effect of LNAME. Mean fractional change in Hbt, Hbo, and Hbr in arteriolar ROI in response to 2 s optogenetic stimulation of nNOS-INs (top row) or 2 s whisker stimulation (lower row) at different time points relative to injection of LNAME. Column 1: experiment immediately prior to LNAME injection; Column 2: experiment immediately following LNAME injection; Column 3: experiment commencing 60 to 70 min post LNAME injection; Column 4: experiment commencing 95 to 135 min post LNAME injection. Blue shading indicates photostimulation period (top row), grey shading indicates whisker stimulation period (lower row). Data are mean ± SEM, n indicates number of mice. Note: the following data are also included in Fig. 1: pre-LNAME; post-LNAME (95 to 135 min [n=9] and 60 to 70 min [n=3]).

Seventy minutes was found to be sufficient time for a significant effect of LNAME to be observed (Fig. 2; F(1.335,14.685)=21.442, p=0.00015, $\eta^{2}=0.661$, n=12; pre-LNAME versus 60 to 70 min, p=0.0005; see Table S2 in the Supplementary Material for all pairwise comparisons). Therefore, post-LNAME hemodynamic measurements were obtained 70 to 135 min after systemic injection of LNAME. Following treatment with LNAME, the nNOS IN-evoked hemodynamic response was inverted, showing a decrease in Hbt and Hbo and an increase in Hbr [Fig. 1(a)]. Inspection of the timeseries of the hemodynamic response [Fig. 1(f)] revealed a greater reduction in Hbt during the photostimulation period (compared with pre-LNAME), followed by an increase in Hbt which peaked after stimulation offset [Figs. 1(c) and 1(f)]. By contrast, the initial hemodynamic response evoked by whisker stimulation was unchanged by LNAME in terms of polarity and timing [Fig. 1(d), Table S3 in the Supplementary Material]. The localized hemodynamic response to whisker stimulation consisted of an increase in Hbt during the stimulation period, which peaked after stimulation offset, both before and after treatment with LNAME [Figs. 1(e) and 1(f)].

**Fig. 3:**
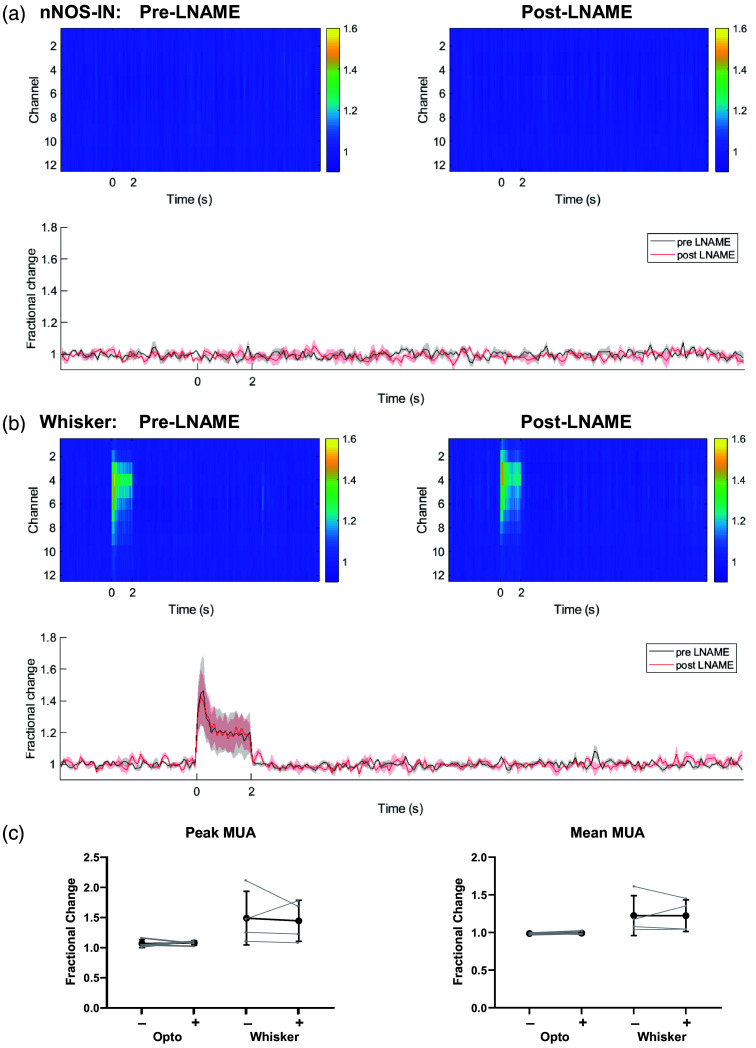
Stimulation-evoked multi-unit activity (MUA) is unchanged in presence of LNAME. Neural responses to (a) 2 s optogenetic stimulation of nNOS-INs or (b) 2 s whisker stimulation (stimulations start at 0 s). Top row: mean change in MUA compared with baseline throughout cortical depth (as indicated by electrode channel number). Color bar represents fractional change. Left: pre-LNAME injection. Right: post-LNAME injection. Bottom row: mean time series of response taken from channels 3 to 6 of electrode (mean ± SEM). Data were collected concurrently with subset of hemodynamic responses displayed in Fig. 1. (c) Peak and mean MUA during 2 s optogenetic or whisker stimulation with (+) and without (−) LNAME. Darker lines represent group mean ± SD, lighter lines indicate trial-averaged means for individual animals. n=4 mice for all panels.

A subset of animals underwent acute implantation of an electrode, to allow concurrent measurement of evoked hemodynamic (Fig. 1) and neural (Fig. 3) changes. As electrode placement elicits a CSD, which can have long-lasting confounding effects on hemodynamic measures,^59^^,^^60^ insertion of an electrode was included as a factor in the statistical analysis of the hemodynamic data. Therefore, a three-way mixed ANOVA was used to assess the effect of LNAME, stimulation type, and electrode insertion on the evoked hemodynamic response. Due to the bidirectional nature of the nNOS IN-evoked hemodynamic response, three metrics were analyzed [Fig. 1(g)], (1) maximal change during initial response [“initial minima,” occurring between 0.25 and 5s after stimulation onset, Fig. 1(h)], (2) maximal change during later response [“peak,” occurring between 0.25 and 10s after stimulation onset, Fig 1(i)], and (3) peak-to-peak amplitude [calculated as (peak-initial minima), Fig. 1(j)]. For all hemodynamic profiles (Hbt, Hbo, and Hbr), for all metrics considered, no significant effect of electrode insertion was found (Tables S4–S6 in the Supplementary Material); therefore, hemodynamic data from all mice were combined (Fig. 1). This lack of effect of CSD on hemodynamic responses is likely due to the fact that electrode insertion occurred ∼50  min prior to collection of pre-LNAME data, sufficient time for hemodynamic recovery after CSD.^51^^,^^60^ As a statistically significant interaction between stimulation type and LNAME was revealed for all hemodynamic profiles (initial minima: Table S4 in the Supplementary Material, peak: Table S5 in the Supplementary Material), suggesting that the effect of LNAME depends on the type of stimulation applied, simple effects tests to assess the effect of LNAME for each stimulation type were performed.

In the presence of LNAME, nNOS IN stimulation evoked a larger “initial minima” [Fig. 1(h), n=12], resulting in a larger initial decrease in Hbt (pre=0.988±0.002, post=0.963±0.006, $p=4.69\times 10^{-4}$) and Hbo (pre=0.977±0.003, post=0.935±0.011, $p=8.28\times 10^{-4}$), and a greater initial increase in Hbr (pre=1.031±0.004, post=1.079±0.014, p=0.002). Furthermore, post LNAME the “peak” hemodynamic response to nNOS IN activation was reduced across all hemoglobin components [Hbt: pre=1.036±0.007, post=1.017±0.005, p=0.013; Hbo: pre=1.064±0.011, post=1.032±0.008, p=0.016; Hbr: pre=0.921±0.012, post=0.953±0.009, p=0.023, Fig. 1(i), n=12], compared with before LNAME injection.

This observed decrease in peak hemodynamic response may be due to an increased initial vasoconstriction [Fig. 1(h)] occurring prior to an unchanged vasodilation. In support of this suggestion, the nNOS IN-evoked peak-to-peak amplitude (measured as minima to peak) in Hbt, Hbo, and Hbr was indeed unchanged in the presence of LNAME [Fig 1(j), Table S6 in the Supplementary Material]. These data suggest that although the initial hemodynamic response to nNOS IN activity is dependent on NO production by NOS, a second, NO-independent, pathway underlies the later increases in Hbt, Hbo, and associated washout of Hbr.

In contrast to the significant effect on the nNOS IN-evoked hemodynamic response, changes in Hbt, Hbo, and Hbr evoked by whisker stimulation were unchanged in the presence of LNAME [Figs. 1(f)–1(j), Tables S4–S6 in the Supplementary Material]. Furthermore, Hbt rise time (time between 10% and 85% of peak response), time to peak, and onset time were all unaffected by LNAME (Table S3 in the Supplementary Material). These data suggest that NO is not involved in the initiation of sensory-evoked functional hyperemia in the somatosensory cortex.

The emergence of an oscillation in all hemodynamic components on return to baseline following stimulation in the presence of LNAME was notable in both photostimulation- and whisker stimulation-evoked responses [Fig. 1(f)], suggesting that NO may play a role in damping the hemodynamic return to baseline following either nNOS IN or whisker stimulation.

**Fig. 4:**
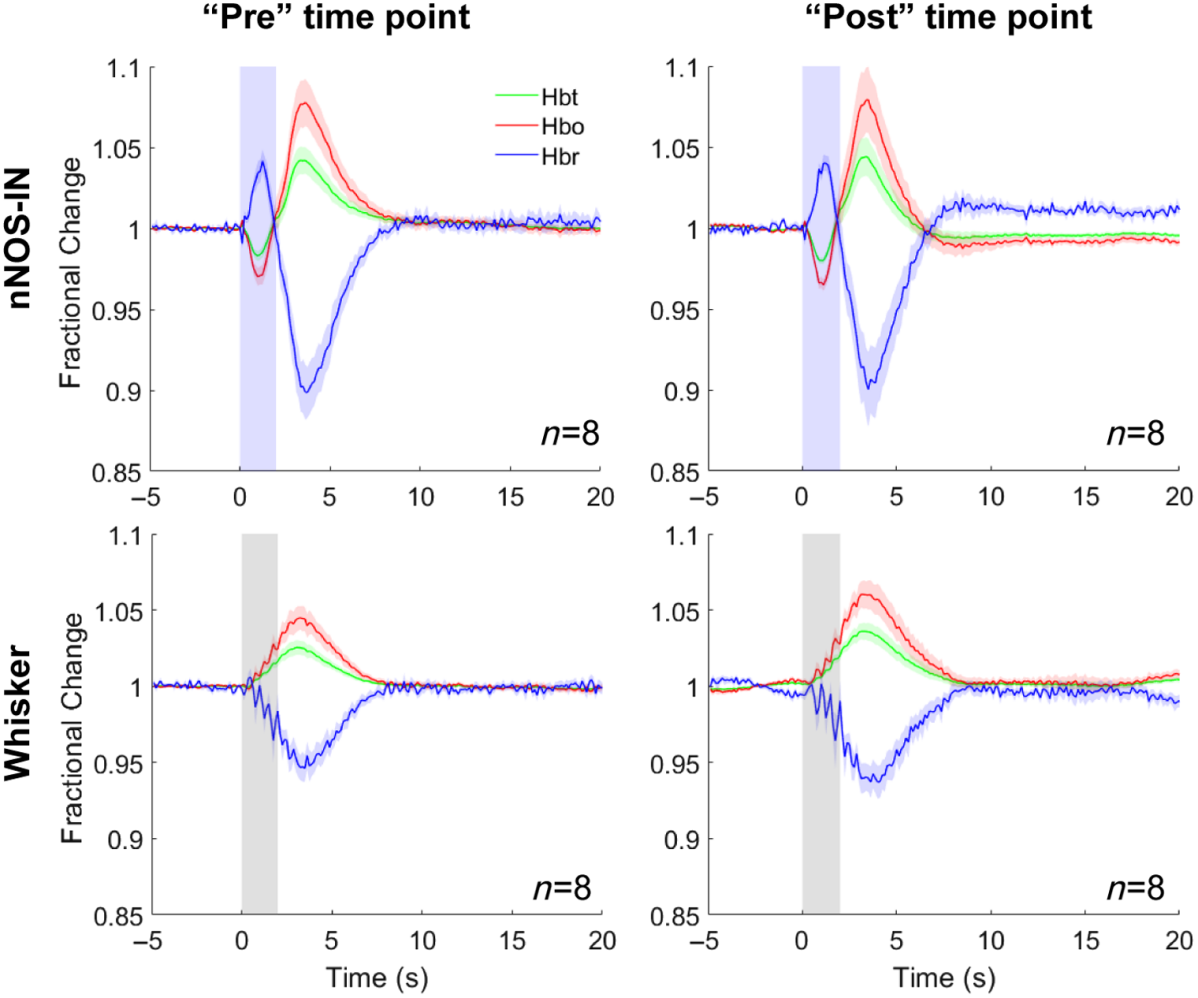
Time-matched experiments with no inhibitor applied. Mean fractional change in Hbt, Hbo, and Hbr in arteriolar ROI in response to 2 s optogenetic activation of nNOS-INs (top row) or 2 s whisker stimulation (bottom row) in anaesthetized mice in which no pharmacological inhibitor was applied. The “pre” (left) and “post” (right) measurement points were time-matched to the pre and post time points in the experiments in which LNAME was administered (Fig. 1). Blue shading indicates photostimulation period (top row), grey shading indicates whisker stimulation period (bottom row). Data are mean ± SEM, n represents number of mice.

To confirm that the observed LNAME-associated differences in nNOS IN-evoked hemodynamic changes were not due to factors such as duration of imaging under anesthesia, in a subset of anesthetized mice, time-matched experiments were also performed without application of LNAME. No time-associated significant differences were observed in hemodynamic responses to either photostimulation or whisker stimulation, confirming that the described changes are not due to factors associated with the duration of the experiment (Fig. 4, Table S7 in the Supplementary Material).

Although sex hormones, including estrogen, have previously been suggested to interact with NO signaling and vascular tone,^61^ we found no effect of sex on the hemodynamic response to either photostimulation of nNOS INs or whisker stimulation, either in the absence or presence of LNAME (Fig. S1 and Table S8 in the Supplementary Material).

Taken together, these data suggest that although NO plays a key role in the initial hemodynamic response evoked by nNOS IN activation, the initiation of whisker stimulation-evoked hemodynamic changes is largely independent of NOS activity.

### 3.2 Evoked Neural Activity Was Unaltered by NOS Inhibition

As the measured hemodynamic responses reflect stimulation-evoked neural activity, we assessed whether LNAME alters evoked neural activity. In a subset of mice, stimulation-evoked electrophysiological and hemodynamic changes were measured concurrently before and after LNAME injection to confirm that the observed LNAME effects reflect altered vascular responses, rather than a change in evoked neural activity. A 16-channel Neuronexus electrode was inserted into the center of the whisker barrel cortex to measure the electrophysiological response to stimulation. In agreement with our previous findings,^7^ 2 s photostimulation of cortical nNOS INs evoked a robust hemodynamic response (Fig. 1) in the absence of a measurable change in ongoing neural activity [multi-unit activity (MUA); Fig. 3(a)]. The lack of measurable change in neural activity persisted in the presence of LNAME [Figs. 3(a) and 3(c)]. Similarly, whisker stimulation-evoked increases in MUA, which extended throughout the depth of the cortex and lasted for the duration of the stimulation [Fig. 3(b)], were unaffected by LNAME [Figs. 3(b) and 3(c)]. These data confirm that LNAME had no effect (peak MUA: F(1,3)=0.032, p=0.869; mean MUA: F(1,3)=0.003, p=0.958, n=4; Table S9 in the Supplementary Material) on the neural activity underlying the evoked localized hemodynamic responses (Fig. 1).

### 3.3 NO Reduces 20-HETE-Evoked Vasoconstriction During nNOS IN Activation

**Fig. 5:**
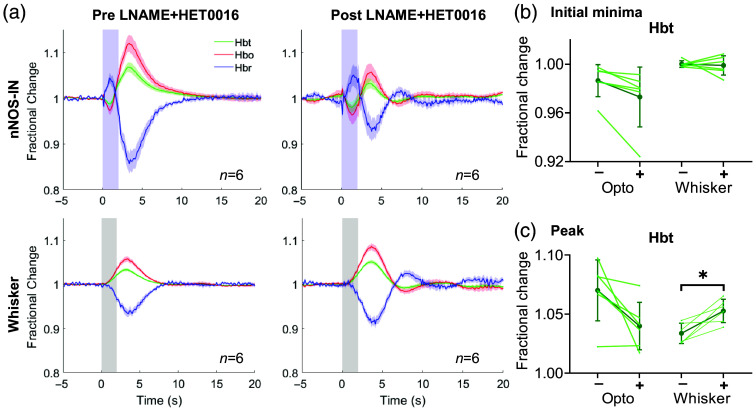
Hemodynamic responses evoked by nNOS-IN activation or whisker stimulation in presence of simultaneous inhibition of NOS and 20-HETE synthesis. Group data (n=6 mice). (a): Mean fractional change in Hbt, Hbo, and Hbr in arteriolar ROI before (left) and after (right) LNAME and HET0016 injection. Blue shading indicates photostimulation period (upper panels), grey shading indicates whisker stimulation period (lower panels). Data are mean ± SEM. (b), (c) Darker lines represent group mean ± *SD*, lighter lines indicate trial-averaged mean for individual animals. (b) Initial fractional change (“initial minima”) in Hbt in response to optogenetic and whisker stimulation, with (+) and without (−) inhibitors (LNAME+HET0016). (c) Maximum fractional change in Hbt (“peak”) evoked by optogenetic and whisker stimulation, with (+) and without (−) LNAME+HET0016. *p<0.025.

As NO is known to inhibit 20-HETE formation,^62^ we hypothesized that the larger initial decrease in Hbt (indicative of vasoconstriction) observed in response to nNOS IN activation [Figs. 1(a), 1(c), 1(f), and 1(h)] in the presence of the NOS inhibitor LNAME was due to 20-HETE production.^63^ To further investigate the NOS-dependent vasoactive pathway underlying nNOS IN-evoked localized hemodynamic changes, in a separate cohort of mice, nNOS IN and whisker stimulation-evoked hemodynamic changes were recorded before and after combined treatment with LNAME (75  mg/kg, i.p.) and HET0016 [a selective inhibitor of CYP4A and CYP4F^48^ which produce 20-HETE, 10  mg/kg, i.v.^49^; Fig. 5]. Repeated measures two-way ANOVAs (factors: drug, stimulation type) revealed interactions between stimulation and drug for both the Hbt initial minima (F(1,5)=18.809, p=0.007, $\eta^{2}=0.790$, n=6; Table S10 in the Supplementary Material) and peak (F(1,5)=23.137, p=0.005, $\eta^{2}=0.822$, n=6; Table S11 in the Supplementary Material). Simple effects tests were therefore performed to assess the combined effect of LNAME and HET0016 on each stimulation type. For 2 s photostimulation of nNOS INs, neither the initial minima in Hbt [Fig. 5(b), pre=0.986±0.005, post=0.973±0.01, p=0.05, Table S10 in the Supplementary Material], nor the peak Hbt [Fig. 5(c), pre=1.07±0.011, post=1.04±0.008, p=0.052, Table S11 in the Supplementary Material] were significantly different before and after treatment with LNAME and HET0016. Combined with the results of treating with LNAME alone (Fig. 1), these data suggest that during short duration nNOS IN activation NO acts, at least in part, to reduce 20-HETE-elicited vasoconstriction.

In response to 2 s whisker stimulation, the peak Hbt change was significantly greater when the production of NO and 20-HETE was inhibited [Fig. 5(c), pre=1.034±0.004, post=1.053±0.004, p=0.013, Table S11 in the Supplementary Material], suggesting that the production of 20-HETE during sensory stimulation causes a constriction, which is overcome by a NO-independent vasodilatory mechanism.

As previously observed in the presence of LNAME alone [Fig. 1(f)], an oscillation in all hemodynamic components was observed on return to baseline following stimulation in the presence of LNAME and HET0016 [Fig. 5(a)], further supporting our suggestion that NO plays a role in damping the hemodynamic return to baseline following nNOS IN activation or sensory stimulation.

If NO release by nNOS INs inhibits 20-HETE formation, it would be expected that applying HET0016 in the presence of this NO release would provide no additional reduction in 20-HETE formation and, therefore, have no effect on the nNOS IN-evoked initial reduction in Hbt. Indeed, simple effects tests (Table S12 in the Supplementary Material) suggest that applying HET0016 alone (10  mg/kg, i.v.) did not significantly alter the initial Hbt minima evoked by photostimulation of nNOS INs (Fig. S2 in the Supplementary Material, pre=0.986±0.008, post=0.982±0.008, p=0.043, n=5). Combining these data with the observed effects of LNAME (Fig. 1) and concurrent LNAME and HET0016 (Fig. 5) suggests that NO inhibits the production of 20-HETE in response to nNOS IN activation.

### 3.4 Hemodynamic Responses to Whisker Stimulation and nNOS IN Activation Sum in a Linear Manner

**Fig. 6:**
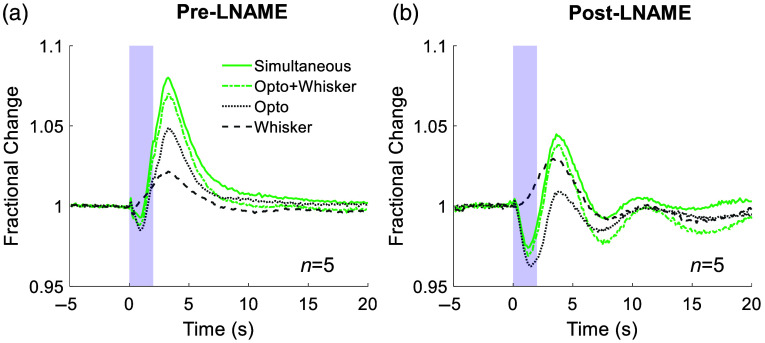
Change in Hbt evoked by simultaneous presentation of whisker and optogenetic stimulation is similar to that predicted by summing changes in Hbt evoked by independent optogenetic and whisker stimulation. Group data (n=5 mice): Mean fractional change in Hbt in arteriolar ROI before (a) and after (b) NOS inhibition with LNAME. Blue shading indicates stimulation period. Continuous green line indicates hemodynamic response to 2 s simultaneous stimulation, and dash and dot green line indicates the predicted response calculated by summing the responses to separate 2 s optogenetic (grey dotted line) and 2 s whisker (grey dashed line) stimulations. For visual clarity, error bars are not shown. (Separate optogenetic and whisker stimulation data are also included in Fig. 1.)

Having demonstrated the differential involvement of NO and 20-HETE in the hemodynamic responses to nNOS IN activation and whisker stimulation, we hypothesized that whisker stimulation-evoked functional hyperemia and nNOS IN-evoked hemodynamic responses are driven by different vasoactive pathways. If this is the case, summing the change in Hbt evoked by photostimulation of cortical nNOS INs and that evoked by whisker stimulation should predict the change in Hbt evoked by simultaneous presentation of whisker and nNOS IN stimulation. To test this, a subset of animals received simultaneous photostimulation of nNOS INs and whisker stimulation, in addition to the separate photostimulation and whisker stimulation described above. In these mice, linear summation of the Hbt time series evoked by whisker stimulation and that evoked by photostimulation of cortical nNOS INs predicted the time series of the Hbt changes evoked by simultaneous stimulation of nNOS INs and whiskers, both in the absence and presence of LNAME (Fig. 6). This linear summation further supports our suggestion that nNOS IN activation and whisker stimulation drive hemodynamic changes via different vasoactive pathways.

### 3.5 Systemic Injection of LNAME Enhances Vasomotion in Anesthetized Mice

**Fig. 7:**
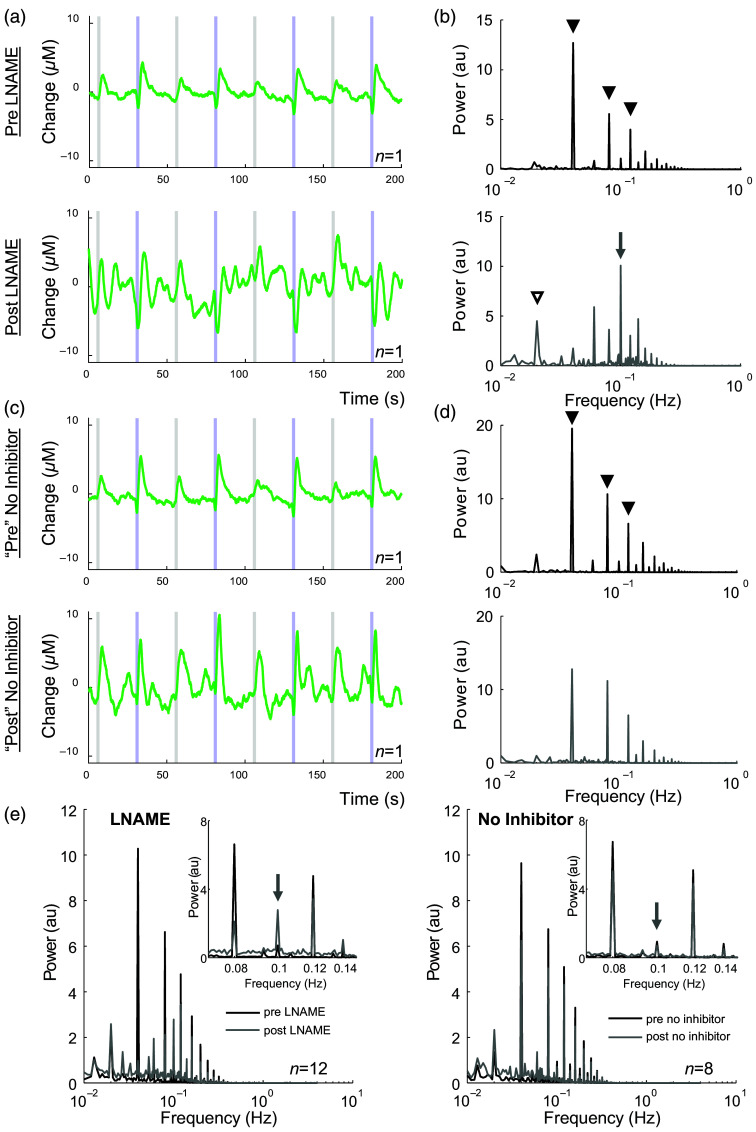
LNAME enhances vasomotion. (a), (c) Example 200 s Hbt time series from experiments occurring before (upper) and after (lower) LNAME injection (a) or matched timepoints with no inhibitor (c). Responses to individual stimulations can be seen. Grey shaded region indicates whisker stimulation, and blue shaded region indicates optogenetic stimulation. (b), (d) Example power spectrum of Hbt data (1000 s duration analyzed) from same experiments as (a), (c), before (upper, black) and after (lower, grey) LNAME injection (b) or matched timepoints with no inhibitor (d). Peaks are observed at frequency of stimulation (ISI 25s: 0.04 Hz) and its harmonics (black arrowheads). After LNAME injection, a peak at 0.1Hz (vasomotion, grey arrow) is observed and the apparent stimulation frequency is reduced (white arrowhead) (e): Mean power spectrum of oscillations in Hbt before (black) and after (grey) LNAME (left, n=12) or no inhibitor (right, n=8). Inset: highlight of 0.07 to 0.15 Hz. Data from arteriolar ROI in experiments as shown in Figs. 1 and 4. For visual clarity, error bars are not shown. n indicates the number of mice.

Vasomotion, a low-frequency oscillation in arteriole diameter occurring at ∼0.1  Hz,^64^^,^^65^ emerges when NOS is inhibited.^66^ Therefore, in addition to characterizing the effect of NOS inhibition by LNAME on stimulation-evoked cortical hemodynamics, we also examined the effect of NOS inhibition on low-frequency arterial oscillations. We detected an increase in the power of low-frequency arterial oscillations, centered around 0.1 Hz, after LNAME injection compared with before injection (Fig. 7, area under the curve (AUC): [0.09 to 0.11 Hz]; p=0.000062, n=12, Tables S13 and S14 in the Supplementary Material), which was not apparent in the absence of LNAME (Fig. 7, “pre” versus “post” in no inhibitor condition, p=0.531, n=8, Tables S13 and S14 in the Supplementary Material). These data confirm that NOS inhibition results in enhanced low frequency vascular oscillations.^31^^,^^67^ Additional peaks in the power spectrum which reflect the frequency of stimulation (ISI of 25s: 0.04 Hz) and its harmonics can be seen in all cases (Fig. 7). As stimulation paradigms were interleaved and LNAME alters the hemodynamic response to photostimulation of nNOS INs but not whisker stimulation (Fig. 1), the peak associated with the stimulation pattern is shifted to a lower frequency in the presence of LNAME [Fig. 7(b)].

### 3.6 Optogenetic Stimulation of nNOS INs Evokes Nonconducted Blood Volume Responses Prior to LNAME Application

**Fig. 8:**
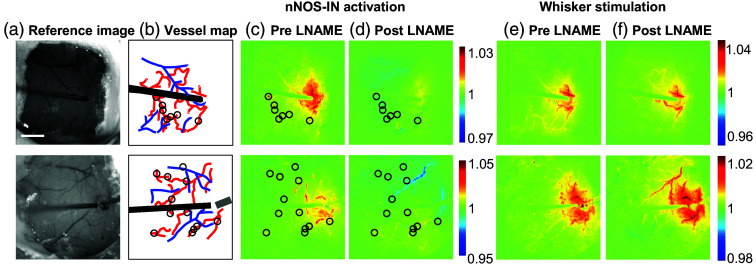
Optogenetic nNOS-IN stimulation produces nonconducted increases in Hbt across the arterial vascular network. (a) Reference images from two representative animals. Scalebar represents 1 mm. (b) Vessel map diagram and location of optical probe (black rectangle) and recording electrode (grey rectangle). (c)–(f) Average Hbt image taken 2 to 5s after the start of optogenetic nNOS-IN stimulation (c)–(d) or whisker stimulation (e)–(f), pre (c) and (e) and post (d) and (f) injection of LNAME. Color bars represent fractional change as compared with baseline. Black circles indicate local, nonconducted blood volume increases.

Following nNOS optogenetic stimulation, in addition to the stereotypical widespread hemodynamic response, we commonly observed an increase in blood volume at specific points along the surface arterial tree [see Figs. 8(a)–8(c), black circles]. After the addition of LNAME, these specific regions of nNOS IN-evoked Hbt increase were not present [Fig. 8(d)]. These data suggest that nNOS IN stimulation caused local nonconducted dilations in the arterial network, possibly through local release of NO. Such nonconducted blood volume increases were not observed following whisker stimulation, either in the absence [Fig. 8(e)] or presence [Fig. 8(f)] of LNAME.

## 4 Discussion and Conclusion

Combining *in vivo* 2D-OIS, electrophysiology, optogenetics, and pharmacology, we investigated the role of NO in nNOS IN driven and sensory-evoked neurovascular coupling in the somatosensory cortex of mice. We demonstrated that: first, localized nNOS IN driven hemodynamic changes are mediated by NO; second, initiation of the hemodynamic response to short duration sensory stimulation is not dependent on NO; and third, NOS blockade causes the emergence of vasomotion in anesthetized mice.

By inhibiting NO synthesis with LNAME, we demonstrated the key role that NO plays in the hemodynamic response to nNOS IN activation (Fig. 1). Inhibiting production of NO by NOS revealed a larger initial decrease in Hbt in response to nNOS IN activation, which was attenuated when the production of NO and 20-HETE was inhibited simultaneously. However, following initiation of the hemodynamic response, the successive increase in Hbt was unaltered by NOS inhibition. These data suggest that the initial vasodilatory response elicited by nNOS IN activation is mediated by NO, at least in part by inhibition of constriction by 20-HETE, whereas a later vasodilation involves a NO-independent mechanism. These findings extend previous work in cortical slices suggesting that type II nNOS INs evoke vasodilation by NO release.^68^ A likely candidate for the delayed vasodilation is GABA,^69^^,^^70^ which is released by active nNOS INs and may act either indirectly via astrocytes to elicit hemodynamic changes^71^^,^^72^ or can open chloride ion channels in smooth muscle cells, causing direct dilation of arterioles.^70^

Our demonstration of an nNOS IN-elicited vasoconstriction (which was increased when NOS was inhibited) further supports the idea that arteriolar constrictions may be evoked by INs, rather than by excitatory neurons.^3^ The observed increase in Hbr elicited by nNOS IN activation [Fig. 1(f)] is analogous to the negative BOLD signal, further linking IN activity to negative BOLD fMRI responses.^7^^,^^73^^,^^74^ An initial nNOS IN-evoked Hbt minima was not reported in our previous study.^7^ This is likely due, in part, to the choice of ROI from which data were analyzed. Analyzing our previously acquired data using an arterial ROI (Fig. S3 in the Supplementary Material) revealed hemodynamic responses which display a similar profile to those reported in the current study [Fig. 1(f)].

Although NO was found to mediate nNOS IN driven changes in cerebral blood volume, the neurovascular coupling response to short duration sensory stimulation was largely unaltered when NO production was reduced (Fig. 1). A lack of effect on stimulation-evoked neural activity (Fig. 3) confirmed that sensory-evoked neurovascular coupling was unaffected by treatment with LNAME. Although nNOS- and eNOS-derived NO have been suggested to play a key role in sensory-evoked neurovascular coupling,^22^^,^^34^^,^^36^^,^^75^^,^^76^ our results call into question both the involvement of nNOS INs in sensory-evoked functional hyperemia^21^ and that NO release is an important mediator of sensory-evoked hemodynamic responses.^22^ Our findings are supported by previous studies, which have also reported a limited effect of NOS inhibition^4^^,^^57^^,^^77^^,^^78^ or genetic deletion of nNOS^29^^,^^37^ on sensory-evoked functional hyperemia. Prior experimental^79^ and modelling studies^80^ have suggested potassium release by active neurons as an alternative driver of sensory-evoked functional hyperemia.

Discrepancies in the reported effects of LNAME in the literature may be due to differences in experimental approaches. First, the effect of LNAME may be affected by the application route chosen. Here, we used i.p. injection of LNAME to reduce NOS activity throughout the brain,^47^^,^^52^ as compared with topical application, which may result in effects being restricted to the superficial cortical layers, or intracortical injection which may limit the effect on pial arterioles^4^—the vascular component most affected by NO.^21^^,^^81^ Second, stimulation duration may dictate the effect of NOS inhibition on the evoked hemodynamic response, with opposing effects being previously reported for long (60 s) and short (4 s) duration sensory stimulation.^31^ Therefore, the discrepancy between our results and those of Liu et al. who used a similar pharmacological approach to report a significant involvement of NO (via inhibition of 20-HETE production) in the hemodynamic response to sensory stimulation in rats may be at least partly explained by the use of short (2 s, here) and long (60 s^29^) duration stimulations. This is further supported by a recent report that pharmacological ablation of Type I nNOS INs reduces Hbt responses to prolonged, but not brief, whisker stimulation.^82^

Although we found no evidence for the involvement of NO in the initiation of sensory-evoked functional hyperemia, following the inhibition of NO production, we observed an oscillation in Hbt, Hbo, and Hbr on return to baseline at stimulation offset in both the photostimulation and whisker stimulation experiments [Fig. 1(f)]. The shape of the haemodynamic response indicates that the hemodynamic return to baseline following stimulation is underdamped, suggesting that NO acts to dampen vasoconstriction after stimulation ends.^80^^,^^83^ A similar arteriolar constriction, sometimes seen following sensory stimulation, has been shown to be mediated by NPY.^3^ Future work could elucidate whether NO attenuates the vasoconstrictive actions of NPY release from nNOS INs following sensory stimulation.

In the current study, a common occurrence following nNOS optogenetic stimulation was a nonconducted blood volume increase at specific points along the surface arterial tree [Fig. 8(c), black circles]. This observation suggests that nNOS IN stimulation caused local nonconducted dilations in the arterial network potentially due to local release of NO in close proximity to the vessels. This is supported by Kurjiaka and Segal,^84^ who showed that when a distal portion of the anesthetized rat cremaster muscle arterial network is stimulated by acetylcholine a rapid retrograde wave of dilation occurred. However, in the same preparation, the addition of the NO donor sodium nitroprusside elicited only dilation local to the application area. In our experiments, after the addition of LNAME, these specific regions of nNOS IN-evoked Hbt increase were not present [Fig. 8(d)], suggesting the inhibition of NO release from nNOS neurons at those specific points along the surface arterial tree. Although our results suggest selective targeting of large arteries on the cerebral surface with nitric oxide release in localized areas close to the arterial wall, further experiments are needed to establish whether this also occurs on penetrating arterioles and within the capillary bed. The same analysis of the Hbt spatial response following sensory whisker stimulation [Figs. 8(e) and 8(f)] fails to show the same effect. There were no local, nonconducted, increases in Hbt following whisker stimulation prior to and after LNAME injection. This further supports the argument against the role of nNOS INs or NO in at least the main retrograde dilation of the surface arteries, which is a major component of the neurovascular response function.^79^^,^^85^^,^^86^

In agreement with previous studies,^31^^,^^66^^,^^67^ we demonstrated a role for NO in suppressing vasomotion. When NO production was attenuated with LNAME, we observed an increase in the power of low frequency hemodynamic oscillations centered at ∼0.1  Hz, compared with before injection of LNAME (Fig. 7). LNAME may lead to the emergence of vasomotion via the inhibition of eNOS-derived NO, which has previously been suggested to inhibit voltage gated calcium channels in arterial smooth muscle cells and, thereby, vasomotion.^87^

Our study is not without limitations. Due to the use of a nonspecific NOS inhibitor, it is not possible for us to dissect the involvement of eNOS and nNOS-associated vasoactive mechanisms in the vascular responses we observed. As both the cellular specificity of NOS isoform expression and the isoform-specificity of pharmacological agents have recently been questioned,^21^ designing experiments that allow specific cellular or isoform dissection is difficult.

Systemic injection of LNAME (75  mg/kg, i.p) can evoke increases in blood pressure in anaesthetized mouse.^88^ As changes in arterial blood pressure may confound the observed changes in hemodynamic low-frequency oscillations,^89^ future work should aim to clearly distinguish the effects of inhibition of nNOS activity from those associated with arterial blood pressure changes. Topical application of NOS inhibitors (previously shown to induce vasomotion^78^) could avoid the potential confound of increased arterial blood pressure.

2D-OIS, used in this study to investigate cortical hemodynamics, predominantly reflects changes in hemoglobin concentration in superficial blood vessels. Although previous studies have suggested that NO is preferentially involved in the regulation of pial arterial, as opposed to capillary, diameter,^21^^,^^81^ and penetrating arteries and capillaries display NO-independent whisker pad stimulation-evoked neurovascular coupling,^57^ further studies using approaches such as 2-photon microscopy would allow assessment of whether nNOS INs can evoke capillary dilation. To evoke vasodilation, NO released by nNOS INs must diffuse to the vessels;^90^ therefore, the regulation of CBF by nNOS INs may depend on their proximity to the vasculature (although recent evidence argues against this^91^). Future assessment of the spatial distribution of nNOS INs in relation to different cerebral vessels (pial arterioles, penetrating arterioles, capillaries) may reveal a potential for nNOS INs to be differentially involved in blood flow control at different points in the vascular tree,^19^ as previously suggested for other cell types.^92^

Anesthesia allows the involvement of NO in the regulation of CBF to be assessed in the absence of behaviors such as locomotion, which has been shown to evoke NO-dependent vasodilation^21^ and can significantly alter sensory stimulus-evoked hemodynamic responses.^55^ Furthermore, the prolonged temporal nature of the experimental paradigm used in this study makes it difficult to perform in awake animals. However, anesthesia may confound neurovascular coupling^93^^,^^94^ and may affect the recruitment of INs to sensory stimulation,^4^ thereby potentially altering the reliance of functional hyperemia on NO or nNOS INs.^91^ To minimize the confound of anesthesia, we used an anesthetic regime under which hemodynamic responses are similar to those observed in the awake mouse.^43^ Furthermore, to compensate for some side effects of anesthesia, mice breathed supplemental oxygen (100%), which likely resulted in hyperoxic conditions.^95^ As hyperoxia can influence NOS activity,^96^ future experiments should explore the oxygen-dependence of the role of nNOS in sensory-evoked functional hyperemia.

Understanding how, and when, nNOS INs, and NO regulate CBF may highlight novel therapeutic strategies for neurodegenerative diseases. CBF deficits are observed early in AD^11^ and are linked to cognitive decline.^97^^,^^98^ Therefore, targeting nNOS INs, or increasing NO bioavailability,^90^ to reverse CBF deficits may prove to be a beneficial strategy for preventing cognitive decline.

## Supplementary Material

10.1117/1.NPh.12.S2. S22802.s01

## Data Availability

Data files generated and analyzed during the current study are available in the DRYAD repository: https://doi.org/10.5061/dryad.jdfn2z3d9.
