# Supplementary material for: Nitric oxide is not responsible for initial sensory-induced neurovascular coupling response in the barrel cortex of lightly anesthetized mice
Source: Neurophotonics. 2025 Jun 20;12(Suppl 2):S22802. doi: 10.1117/1.NPh.12.S2.S22802 (PMC12180670; doi:10.1117/1.NPh.12.S2.S22802)
Supplement: Supplementary file 1 [file NPh_012_S22802_SD001.pdf]

Nitric oxide is not responsible for initial sensory-induced neurovascular coupling response in the barrel cortex of lightly anaesthetised mice.

Llywelyn Lee[1,2,3], Luke W. Boorman[1], Emily Glendenning[1,2,3], Changlin Shen[1], Jason Berwick[1,2,3], Clare Howarth[1,2,3]

[1]Department of Psychology, University of Sheffield, Sheffield, S1 4DP, UK

[2]Neuroscience Institute, University of Sheffield, Sheffield, UK

[3]Healthy Lifespan Institute (HELSI), University of Sheffield, Sheffield, UK

Address correspondence to Dr Clare Howarth, Department of Psychology, University of Sheffield, ICOSS Building, 219 Portobello, Sheffield, UK, S1 4DP.

Email: c.howarth@sheffield.ac.uk

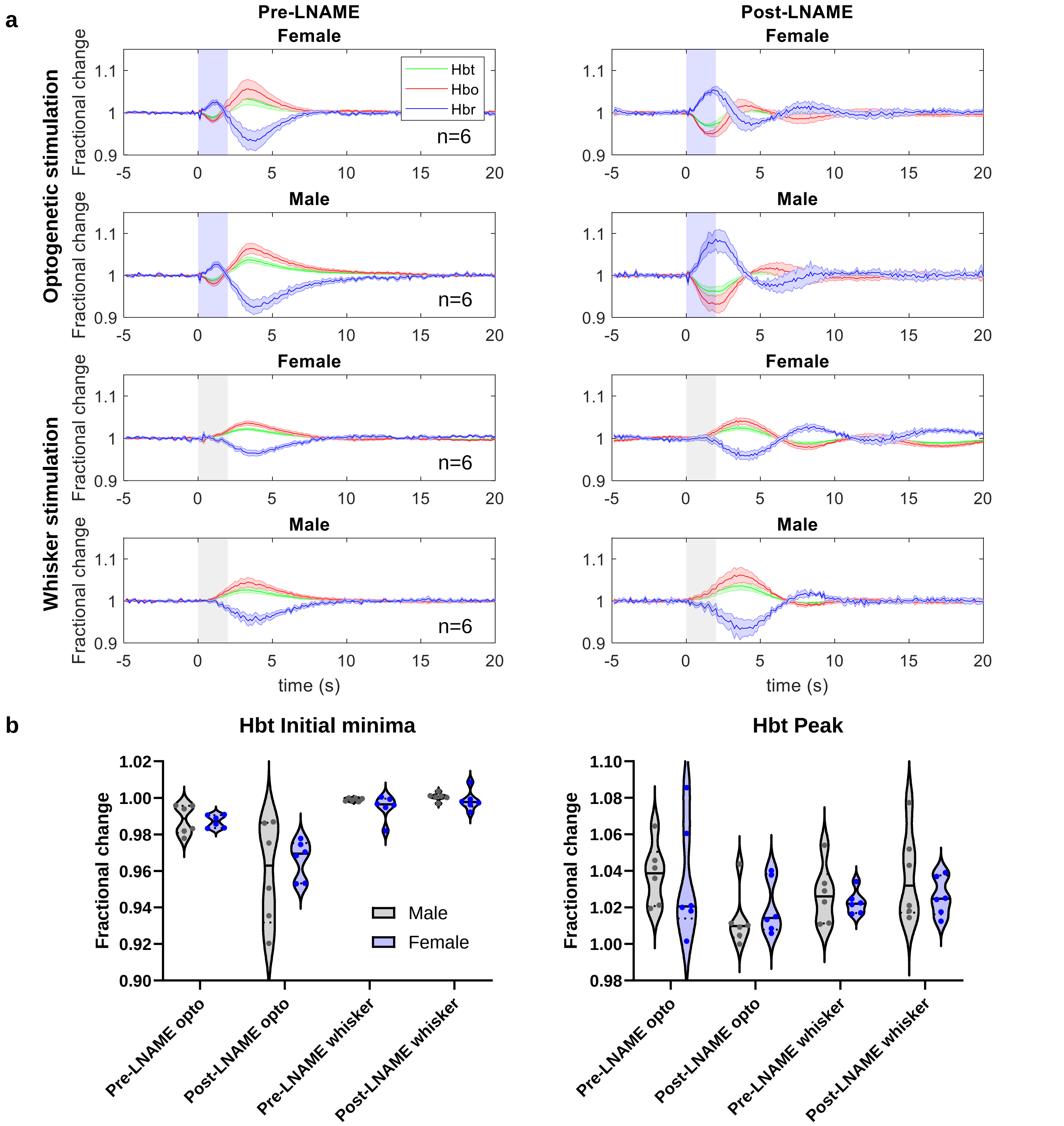

Figure S1: No sex-dependent differences in NOS-dependence of haemodynamic responses evoked by 2s nNOS-IN activation or whisker stimulation. Group data (n=6 female mice and 6 male mice). **(a)** Mean fractional change in Hbt, Hbo and Hbr in arteriolar ROI in response to 2s optogenetic activation of nNOS-INs (top two rows) or 2s whisker stimulation (bottom two rows) before (left) and after (right) LNAME injection. Responses shown for female (higher plots) and male (lower plots) mice. Blue shading indicates photostimulation period (top rows), grey shading indicates whisker stimulation period (bottom rows). Data: mean ± SEM, n represents number of mice. **(b):** Initial fractional change ('initial minima') in Hbt **(Left)** and maximum fractional change in Hbt ('peak', **Right**) evoked by optogenetic and whisker stimulation, before and after LNAME. Solid black line indicates median, dashed black line indicates quartiles. Individual data points are shown. Note: these data are combined in Figure 1.

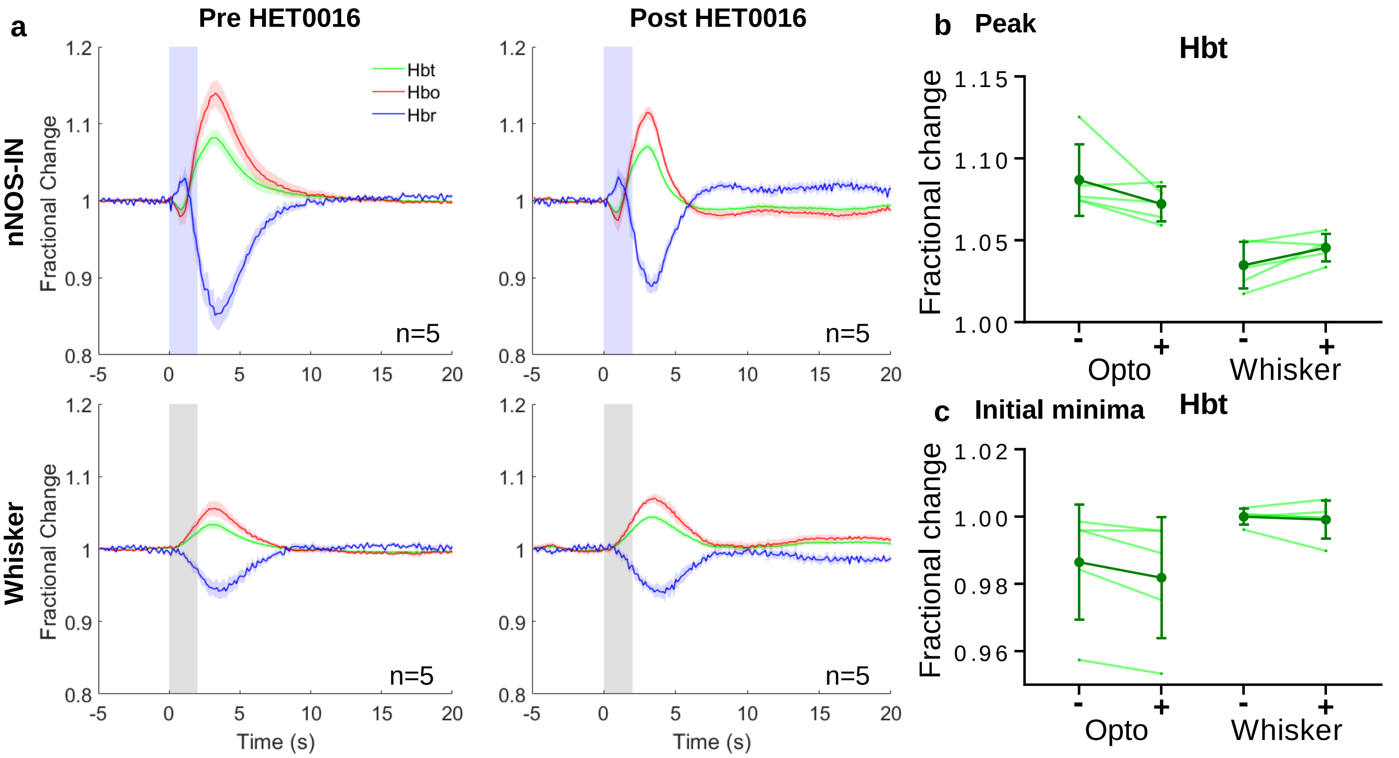

Figure S2: Haemodynamic responses during inhibition of 20-HETE synthesis. (a-c): Group data (n=5 mice). **(a)** Mean fractional change in Hbt, Hbo and Hbr in arteriolar ROI in response to 2s optogenetic activation of nNOS-INs (top row) or 2s whisker stimulation (bottom row) before (left) and after (right) HET0016 injection. Blue shading indicates photostimulation period (top row), grey shading indicates whisker stimulation period (bottom row). Data: mean ± SEM, n represents number of mice. **(b-c):** Darker lines represent group mean ± SD, lighter lines indicate trial-averaged mean for individual animals. **(b):** Maximum fractional change in Hbt (‘peak’) evoked by optogenetic and whisker stimulation, with (+) and without (-) HET0016. **(c):** Initial fractional change (‘initial minima’) in Hbt in response to optogenetic and whisker stimulation, with (+) and without (-) HET0016.

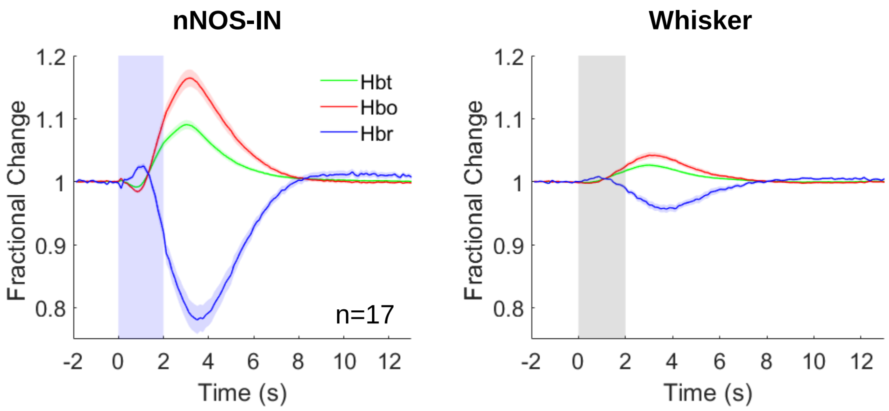

Figure S3: Previously collected[7] haemodynamic responses show biphasic response to photostimulation of nNOS-INs. Mean fractional change in Hbt, Hbo and Hbr in artery ROI in response to 2s, 99Hz optogenetic activation of nNOS-INs (left) or 2s whisker stimulation (right). Blue shading indicates photostimulation period, grey shading indicates whisker stimulation period. Data are mean ± SEM, n represents number of mice. Data are taken from imaging only experiments (i.e. no electrode implantation).

Table S1: Baseline assumptions for artery ROI for spectral analysis of post-pharmacological intervention data were amended for each mouse. Group mean ± SEM are reported. Assumption for “pre” time point is provided for comparison. Hbt: total haemoglobin.

Pharmacological Intervention	Hbt Concentration (µM)	Oxygen Saturation (%)	n (mice)
“Pre” timepoint	100	80	
LNAME	100.534±0.773	79.16±0.006	12
LNAME + HET0016	101.775±1.207	79.23±0.007	6
HET0016	100.836±1.602	80.76±0.005	5
No Inhibitor	100.264±0.729	79.58±0.004	8

Table S2: Pairwise comparisons of total haemoglobin (Hbt) initial minima evoked by nNOS interneuron activation at different time points relative to LNAME injection (Bonferroni adjusted p values reported). (n= 12 mice) *p<0.05, **p<0.01, ***p<0.001

Pairwise comparison	p
Pre-LNAME vs 0mins	0.016*
Pre-LNAME vs 60-70mins	0.0005***
0mins vs 60-70mins	0.009**

Table S3: Temporal characteristics of whisker stimulation-evoked haemodynamic response (total haemoglobin, Hbt), before and after LNAME injection.

Parameter	Pre-LNAME [mean ± s.d]	Post-LNAME [mean ± s.d]	Paired t-test	n (mice)
Rise time (s)	1.76 ± 0.29	1.88 ± 0.35	$t(11) = -0.87$, $p = 0.403$	12
Time to peak (s)	2.63 ± 0.32	3.01 ± 0.63	$t(11) = -1.92$, $p = 0.082$	12
Onset time (s)	0.63 ± 0.21	0.77 ± 0.60	$t(11) = -0.77$, $p = 0.461$	12

Table S4: Effect of LNAME on haemodynamic response – Initial minima: 3-way mixed ANOVA results. Bonferroni correction for multiple comparisons was applied, to account for three different haemodynamic profiles (total haemoglobin: Hbt, oxyhaemoglobin: Hbo, and deoxyhaemoglobin: Hbr). (n= 12 mice) *p<0.017, **p<0.003, *** p<0.0003. Significant interactions were followed up using simple effects tests (*p<0.025, **p<0.005, ***p<0.0005, following Bonferroni correction for multiple comparisons).

Variable	Factor	F(1,10)		
		F	P	η^2
Hbt response in initial minima period	Electrode	1.208	0.297	0.108
	Stim	31.075	0.000***	0.757
	Stim*Electrode	0.572	0.467	0.054
	Drug	21.2	0.001**	0.679
	Drug*Electrode	0.001	0.982	0.000
	Stim*Drug	25.563	0.000**	0.719
	Stim*Drug*Electrode	0.137	0.719	0.014
Hbo response in initial minima period	Electrode	0.897	0.366	0.082
	Stim	31.342	0.000***	0.758
	Stim*Electrode	0.383	0.55	0.037
	Drug	17.618	0.002**	0.638
	Drug*Electrode	0.000	0.994	0.000
	Stim*Drug	22.81	0.001**	0.695
	Stim*Drug*Electrode	0.024	0.881	0.002
Hbr response in initial minima period	Electrode	0.282	0.607	0.027
	Stim	27.526	0.000**	0.734
	Stim*Electrode	0.014	0.908	0.001
	Drug	13.484	0.004*	0.574
	Drug*Electrode	0.018	0.896	0.002
	Stim*Drug	16.223	0.002**	0.619
	Stim*Drug*Electrode	0.036	0.853	0.004
Simple effects tests: LNAME (2s whisker stimulation)			p	
Hbt response in initial minima period			0.159	
Hbo response in initial minima period			0.201	
Hbr response in initial minima period			0.434	

Table S5: Effect of LNAME on haemodynamic response – Peak: 3-way mixed ANOVA results. Bonferroni correction for multiple comparisons was applied, to account for three different haemodynamic profiles (total haemoglobin: Hbt, oxyhaemoglobin: Hbo, and deoxyhaemoglobin: Hbr). (n= 12 mice) *p<0.017, **p<0.003, *** p<0.0003. Significant interactions were followed up using simple effects tests (*p<0.025, **p<0.005, ***p<0.0005, following Bonferroni correction for multiple comparisons).

Variable	Factor	F(1,10)		
		F	P	η^2
Peak Hbt response	Electrode	2.058	0.182	0.171
	Stim	0.085	0.776	0.008
	Stim*Electrode	0.010	0.924	0.001
	Drug	3.058	0.111	0.234
	Drug*Electrode	2.851	0.122	0.222
	Stim*Drug	9.229	0.013*	0.48
	Stim*Drug*Electrode	1.450	0.256	0.127
Peak Hbo response	Electrode	1.966	0.191	0.164
	Stim	0.008	0.931	0.001
	Stim*Electrode	0.015	0.905	0.001
	Drug	2.287	0.161	0.186
	Drug*Electrode	2.533	0.143	0.202
	Stim*Drug	8.795	0.014*	0.468
	Stim*Drug*Electrode	1.433	0.259	0.125
Peak Hbr response	Electrode	2.079	0.180	0.172
	Stim	0.312	0.589	0.030
	Stim*Electrode	0.090	0.77	0.009
	Drug	0.981	0.345	0.089
	Drug*Electrode	1.641	0.229	0.141
	Stim*Drug	8.551	0.015*	0.461
	Stim*Drug*Electrode	1.744	0.216	0.149
Simple effects tests: LNAME (2s whisker stimulation)			p	
Peak Hbt response			0.129	
Peak Hbo response			0.124	
Peak Hbr response			0.127	

Table S6: Effect of LNAME on haemodynamic response – peak-to-peak amplitude: 3-way mixed ANOVA results. Bonferroni correction for multiple comparisons was applied, to account for three different haemodynamic profiles (total haemoglobin: Hbt, oxyhaemoglobin: Hbo, and deoxyhaemoglobin: Hbr). (n=12 mice) *p<0.017, **p<0.003, ***p<0.0003.

Variable	Factor	F(1,10)		
		F	p	η^2
Peak-to-peak amplitude: Hbt	Electrode	2.118	0.176	0.175
	Stim	12.499	0.005*	0.556
	Stim*Electrode	0.408	0.537	0.039
	Drug	1.489	0.25	0.13
	Drug*Electrode	1.296	0.281	0.115
	Stim*Drug	0.079	0.784	0.008
	Stim*Drug*Electrode	0.943	0.354	0.086
Peak-to-peak amplitude: Hbo	Electrode	1.805	0.209	0.153
	Stim	15.061	0.003*	0.601
	Stim*Electrode	0.334	0.576	0.032
	Drug	1.784	0.211	0.151
	Drug*Electrode	1.06	0.327	0.096
	Stim*Drug	0.114	0.743	0.011
	Stim*Drug*Electrode	1.253	0.289	0.111
Peak-to-peak amplitude: Hbr	Electrode	1.334	0.275	0.118
	Stim	19.48	0.001**	0.661
	Stim*Electrode	0.163	0.695	0.016
	Drug	2.181	0.171	0.179
	Drug*Electrode	0.749	0.407	0.07
	Stim*Drug	0.234	0.639	0.023
	Stim*Drug*Electrode	2.321	0.159	0.188

Table S7: Results of 2-way repeated measures ANOVA comparing haemodynamic responses (total haemoglobin: Hbt) in no inhibitor condition (n=8 mice, *p<0.05, **p<0.01, ***p<0.001), and simple effects tests (following Bonferroni correction for multiple comparisons, *p<0.025, **p<0.005, ***p<0.0005).

Variable	Factor	F(1,7)		
		F	p	η^2
Hbt response in initial minima period	Stim	106.469	0.000017***	0.938
	Timepoint	0.791	0.403	0.102
	Stim*Timepoint	7.450	0.029*	0.516
Peak Hbt response	Stim	2.895	0.133	0.293
	Timepoint	1.946	0.206	0.218
	Stim*Timepoint	1.133	0.322	0.139
Peak-to-peak amplitude: Hbt	Stim	14.348	0.007**	0.672
	Timepoint	1.866	0.214	0.21
	Stim*Timepoint	0.113	0.747	0.016

Simple effects tests (Hbt response in initial minima period)		p
Whisker stimulation	‘Pre’ vs ‘Post’	0.183
Optogenetic stimulation	‘Pre’ vs ‘Post’	0.074

Table S8: Effect of sex on haemodynamic response (total haemoglobin, Hbt) to nNOS IN activation or whisker stimulation, before and after LNAME injection. (n = 12 mice).

Parameter	Group	Male n=6<br>[mean ± s.d.]	Female n=6<br>[mean ± s.d.]	Independent samples t-test
Hbt initial minima	Pre-LNAME optogenetic stimulation	0.988 ± 0.008	0.987 ± 0.003	t(6.786) = 0.274, p = 0.792
	Post-LNAME optogenetic stimulation	0.959 ± 0.028	0.966 ± 0.011	t(6.417) = -0.578, p = 0.583
	Post-LNAME whisker stimulation	1.001 ± 0.002	0.999 ± 0.006	t(10) = 0.799, p = 0.221
Hbt peak	Pre-LNAME optogenetic stimulation	1.038 ± 0.017	1.034 ± 0.032	t(10) = 0.242, p = 0.814
	Pre-LNAME whisker stimulation	1.027 ± 0.016	1.023 ± 0.006	t(10) = 0.596, p = 0.564
	Post-LNAME whisker stimulation	1.038 ± 0.025	1.026 ± 0.011	t(6.775) = 1.080, p = 0.317
Parameter	Group	Male n=6<br>Median	Female n=6<br>Median	Mann-Whitney U test
Hbt initial minima	Pre-LNAME whisker stimulation	0.999	0.996	U = 9, z = -1.441, p = 0.150
Hbt peak	Post-LNAME optogenetic stimulation	1.010	1.014	U = 24, z = 0.961, p = 0.337

Table S9: Results of 2-way repeated measures ANOVA for stimulation-evoked multi-unit activity (MUA) in absence and presence of LNAME (n=4 mice).

Variable	Factor	F(1,3)		
		F	p	η^2
MUA Peak	Stim	5.361	0.104	0.641
	Drug	0.032	0.869	0.011
	Drug*Stim	0.245	0.655	0.075
Mean MUA	Stim	3.865	0.144	0.563
	Drug	0.003	0.958	0.001
	Drug*Stim	0.007	0.939	0.002

Table S10: Effect of LNAME and HET0016 on haemodynamic response – Initial minima: 2-way repeated measures ANOVA results (n=6 mice, *p<0.05, **p<0.01, ***p<0.001), and simple effects tests (following Bonferroni correction for multiple comparisons, *p<0.025, **p<0.005, ***p<0.0005). Hbt: total haemoglobin.

Variable	Factor	F(1,5)		
		F	p	η^2
Hbt response in initial minima period	Stim	7.549	0.04*	0.602
	Inhibitor	2.697	0.161	0.350
	Stim*Inhibitor	18.809	0.007**	0.790
Simple effects tests				
Whisker stimulation	'Pre' vs 'Post'		0.785	
Optogenetic stimulation	'Pre' vs 'Post'		0.050	

Table S11: Effect of LNAME and HET0016 on haemodynamic response – Peak: 2-way repeated measures ANOVA results (n=6 mice, *p<0.05, **p<0.01, ***p<0.001), and simple effects tests (following Bonferroni correction for multiple comparisons, *p<0.025, **p<0.005, ***p<0.0005).

Hbt: total haemoglobin.

<table>
<tr><th rowspan="2">Variable</th><th rowspan="2" colspan="2">Factor</th><th colspan="3">F(1,5)</th></tr>
<tr><th>F</th><th>p</th><th>η^2</th></tr>
<tr><td rowspan="3">Peak Hbt response</td><td colspan="2">Stim</td><td>2.776</td><td>0.157</td><td>0.357</td></tr>
<tr><td colspan="2">Drug</td><td>0.548</td><td>0.492</td><td>0.099</td></tr>
<tr><td colspan="2">Stim*Drug</td><td>23.137</td><td>0.005**</td><td>0.822</td></tr>
<tr><td colspan="4">Simple effects tests</td><td colspan="2">p</td></tr>
<tr><td colspan="2">Whisker stimulation</td><td colspan="2">‘Pre’ vs ‘Post’</td><td colspan="2">0.013*</td></tr>
<tr><td colspan="2">Optogenetic stimulation</td><td colspan="2">‘Pre’ vs ‘Post’</td><td colspan="2">0.052</td></tr>
</table>

Table S12: Effect of HET0016, results of 2-way repeated measures ANOVA comparing haemodynamic responses (n=5 mice), *p<0.05, **p<0.01, ***p<0.001, and simple effects tests (following Bonferroni correction for multiple comparisons, *p<0.025, **p<0.005, ***p<0.0005). Hbt: total haemoglobin.

Variable	Factor	F(1,4)		
		F	p	η^2
Hbt response in initial minima period	Stim	4.220	0.109	0.513
	Drug	3.336	0.142	0.455
	Stim*Drug	28.469	0.006**	0.877
Peak Hbt response	Stim	30.061	0.005**	0.883
	Drug	0.225	0.660	0.053
	Stim*Drug	5.911	0.072	0.596

Hbt minima: Simple effects tests		p
Optogenetic stimulation	‘Pre’ vs ‘Post’	0.043
Whisker stimulation	‘Pre’ vs ‘Post’	0.587

Table S13: Descriptive statistics of power in low frequency arteriole oscillations (Area under curve (AUC), 0.09-0.11Hz).

Variable		Mean	SEM	n (mice)
AUC (0.09-0.11Hz) LNAME	Pre	2.42	0.24	12
	Post	9.80	1.62	
AUC (0.09-0.11Hz) No Inhibitor	Pre	2.70	0.35	8
	Post	3.82	0.54	

Table S14: Statistical analysis of power in low frequency arteriole oscillations: 2 way mixed ANOVA (*p<0.05, **p<0.01, ***p<0.001) and simple effects tests (following Bonferroni correction for multiple comparisons, *p<0.0125, **p<0.0025, ***p<0.00025). AUC: Area under curve.

Variable	Factor	F(1,18)		
		F	p	η^2
AUC (0.09-0.11Hz)	Time	14.265	0.001**	0.442
	Drug	8.948	0.008**	0.332
	Drug*Time	7.765	0.012*	0.301

Simple effects tests		p
LNAME	Post vs Pre	0.000062***
No Inhibitor	Post vs Pre	0.531
Pre timepoint	LNAME vs No Inhibitor	0.497
Post timepoint	LNAME vs No Inhibitor	0.009*
